# Immune Milieu and Genomic Alterations Set the Triple-Negative Breast Cancer Immunomodulatory Subtype Tumor Behavior

**DOI:** 10.3390/cancers13246256

**Published:** 2021-12-13

**Authors:** Rubén Rodríguez-Bautista, Claudia H. Caro-Sánchez, Paula Cabrera-Galeana, Gerardo J. Alanis-Funes, Everardo Gutierrez-Millán, Santiago Ávila-Ríos, Margarita Matías-Florentino, Gustavo Reyes-Terán, José Díaz-Chávez, Cynthia Villarreal-Garza, Norma Y. Hernández-Pedro, Alette Ortega-Gómez, Luis Lara-Mejía, Claudia Rangel-Escareño, Oscar Arrieta

**Affiliations:** 1 Laboratorio de Medicina Personalizada de la Unidad de Oncología Torácica, Instituto Nacional de Cancerología (INCan), Mexico City 14080, Mexico; rubenrb@comunidad.unam.mx (R.R.-B.); nhernandezp@incan.edu.mx (N.Y.H.-P.); 2 Programa de Doctorado en Ciencias Biomédicas, Facultad de Medicina, Universidad Nacional Autónoma de México (UNAM), Mexico City 04510, Mexico; 3 Departmento de Patología, Instituto Nacional de Cancerología (INCan), Mexico City 14080, Mexico; ccavos@incan.edu.mx; 4 Departamento de Oncología Médica-Tumores Mamarios, Instituto Nacional de Cancerología (INCan), Mexico City 14080, Mexico; pcabrerag@incan.edu.mx (P.C.-G.); cynthia.villarreal@tecsalud.mx (C.V.-G.); 5 Bio Assisted Sequencing Environment National Sequencing Laboratory (Tec-BASE), School of Engineering and Sciences, Tecnologico de Monterrey, Monterrey 64849, Mexico; gerardo.alanisf@tec.mx; 6 Centro de Investigación sobre Enfermedades Infecciosas, Instituto Nacional de Salud Pública, Cuernavaca 62100, Mexico; everardo.gutierrez@insp.edu.mx; 7 Centro de Investigación de Enfermedades Infecciosas, Instituto Nacional de Enfermedades Respiratorias, Mexico City 14080, Mexico; santiago.avila@cieni.org.mx (S.Á.-R.); margarita.matias@cieni.org.mx (M.M.-F.); gustavo.reyesteran@salud.gob.mx (G.R.-T.); 8 Unidad de Investigación Biomédica en Cáncer, INCan-Instituto de Investigaciones Biomédicas, UNAM, Mexico City 14080, Mexico; jdiazchavez03@comunidad.unam.mx; 9 Centro de Cáncer de Mama, Hospital Zambrano Hellion, Tecnologico de Monterrey, Monterrey 66278, Mexico; 10 Laboratorio de Medicina Traslacional, Instituto Nacional de Cancerología (INCan), Mexico City 14080, Mexico; aortegag@incan.edu.mx; 11 Thoracic Oncology Unit, Department of Thoracic Oncology, Instituto Nacional de Cancerología (INCan), Mexico City 14080, Mexico; luis.laram@incmnsz.mx; 12 School of Engineering and Sciences, Tecnologico de Monterrey, Epigmenio González 500, San Pablo, Santiago de Querétaro 76130, Mexico; 13 Computational Genomics and Integrative Biology, National Institute of Genomic Medicine (INMEGEN), Periférico Sur 4809 Arenal Tepepan, Mexico City 14610, Mexico

**Keywords:** immunology, molecular subtype, immune checkpoint inhibitors, programmed death-ligand, tumor-infiltrating lymphocytes

## Abstract

**Simple Summary:**

Triple-negative breast cancer (TNBC) is an aggressive and highly heterogeneous breast cancer subtype, both molecular and transcriptomic. Gene expression patterns identified seven TNBC subtypes; basal-like 1 (BL1), basal-like 2 (BL2), immunomodulatory (IM), mesenchymal (M), mesenchymal stem-like (MSL), luminal androgen receptor (LAR), and unstable (UNS). Herein, we contrasted the IM subtype with non-IM TNBC, including clinical, histopathological, and molecular variables. Our results showed that the IM subtype featured an increased FOXP3+ TILs infiltration and a higher CTLA-4 and PD-L1 expression compared with non-IM tumors. Long intergenic non-coding RNAs associated with the immune response through transcriptomic and enrichment analyses characterized the IM-subtype enriched by the β-catenin signaling pathway. Additionally, DNA sequencing identified differences in mutation rates as well as some specific mutations. These results should motivate the design of future clinical trials in which the benefit of immune-based therapy in this subgroup of patients could be evaluated.

**Abstract:**

Triple-negative breast cancer (TNBC) is an aggressive and heterogeneous disease. Seven subtypes have been described based on gene expression patterns. Herein, we characterized the tumor biology and clinical behavior of the immunomodulatory (IM) subtype. Methods: Formalin-fixed paraffin-embedded tumor samples from 68 high-risk (stage III-IV) TNBC patients were analyzed through microarrays, immunohistochemistry, and DNA sequencing. Results: The IM subtype was identified in 24% of TNBC tumor samples and characterized by a higher intratumoral (intT) and stromal (strml) infiltration of FOXP3+ TILs (Treg) compared with non-IM subtypes. Further, PD-L1+ (>1%) expression was significantly higher, as well as CTLA-4+ intT and strml expression in the IM subtype. Differential expression and gene set enrichment analysis identified biological processes associated with the immune system. Pathway analysis revealed enrichment of the β-catenin signaling pathway. The non-coding analysis led to seven Long Intergenic Non-Protein Coding RNAs (lincRNAs) (6 up-regulated and 1 down-regulated) that were associated with a favorable prognosis in the TNBC-IM subtype. The DNA sequencing highlighted two genes relevant to immune system responses: *CTNNB1* (Catenin β-1) and *IDH1*. Conclusion: the IM subtype showed a distinct immune microenvironment, as well as subtype-specific genomic alterations. Characterizing TNBC at a molecular and transcriptomic level might guide immune-based therapy in this subgroup of patients.

## 1. Introduction

Triple-Negative Breast Cancer (TNBC) is a highly aggressive and life-threatening malignancy. It accounts for approximately 10–15% of breast cancers diagnosed worldwide [1], affecting primarily young women, with a higher prevalence among African-American and Hispanic subgroups [2]. TNBC is characterized as a poor-prognosis malignancy. This subtype lacks the expression of estrogen and progesterone receptors, as well as the overexpression of the HER2 protein. Further, this is a genomically and transcriptomically heterogeneous disease, exhibiting molecular diversity, a higher rate of chromosomal translocations and different gene expression patterns [3]. This intrinsic heterogeneity has motivated the search for subtypes within TNBC. One of the most interesting reports in this regard is the TNBC subtype classifier by Lehmann et al. This study described an intrinsic TNBC subtype characterized by upregulated immune responses, immune cell markers, immune transcription factors, and a predominant dysregulation of immune pathways [4]. Since then, the immunomodulatory (IM) subtype has been proposed for individualized treatment by modulating its immune milieu [5].

Immunotherapy has improved the prognosis of many solid neoplasms [6]. TNBC could also benefit from this type of cancer treatment through a profound understanding of how the immune system regulates the tumor microenvironment, identifying the main components regulated by immunotherapy, such as immune cells surrounding the tumor such as tumor-infiltrating lymphocytes (TIL) [7]. TNBC has already shown intense TILs enhancing tumor responses [8,9]. Recent data have demonstrated an upregulation of the tumor-immune system in subjects undergoing chemotherapy by activating CD8+ (Th1) TILs [8], positively correlating with improved responses [10] and even with increased survival [11,12] Similarly, higher CD4-TILs (Th2) have been associated with favorable outcomes [13]. Immune cell subpopulations that predominate in the IM subtype have not been fully addressed as potential factors in the diversity of clinical outcomes [14].

Anti-PD1 (programmed death-1) therapy plus chemotherapy have been incorporated into the therapeutical arsenal of locally advanced, recurrent, or metastatic PD-L1-positive TNBC, showing a progression-free survival benefit in this subgroup of patients [15,16,17]. More recently, immunotherapy has been introduced in earlier stages, increasing pathological complete responses (pCR) in the neoadjuvant setting [18,19]. PDL-1 has been one of the most, or perhaps the most, studied biomarker in the last decade. However, inconsistencies in its results have affected its reliability [20]. In breast cancer, PD-L1 expression has been observed in 20–50% of tumor cells and TILs [21]. Furthermore, the cytotoxic T lymphocyte antigen 4 (CTLA-4) has been studied in TNBC cells due to its critical functional role in TILs, modulating the immune response [22], although its relevance in clinical terms is limited. Some genomic metrics have been correlated with immune-rich TNBCs, in which a reduced number of somatic copy number alterations, as well as reduced clonal heterogeneity, was inversely associated with an immune metagene expression. Moreover, significantly lower mutations and neoantigen counts were associated with an increased T-lymphocyte infiltration [23]. Nonetheless, the precise determination of which TNBC patient subgroups will benefit from treatment with immune checkpoint inhibitors (ICIs) remains a challenge. Focusing on the intrinsic subtypes might lead to more promising results. This study aimed to characterize the immune milieu through genome alterations, patterns in non-coding regions, gene expression analysis, and the clinical behavior of the TNBC-IM subtype in high-risk clinical stage III and IV TNBC patients.

## 2. Materials and Methods

### 2.1. Sample Selection

Formalin-fixed, paraffin-embedded (FFPE) tissue samples from 68 TNBC patients treated at the Breast Cancer Department in the Instituto Nacional de Cancerología of Mexico (INCan), archived from 2007–2010, were considered eligible for this study. All the samples were confirmed as TNBC, estrogen receptor (ER)-negative, progesterone receptor (PR)-negative, and HER-2-negative by immunohistochemistry assessed according to the 2020 ASCO/CAP guidelines using an FDA-approved assay [24]. The histologic subtype and grade of differentiation were determined according to the WHO classification and the Nottingham histologic grading system [24]. The INCan institutional review board approved the study (No. INCAN/CI/200/13INCAN/CEI/224/13/CEI/847). All the patients provided informed consent and the procedures were carried out under the terms of the Helsinki Declaration. Patients were excluded if clinicopathological information or material/sample were not available or were incomplete. Before inclusion, hematoxylin and eosin (H&E)-stained slides from tumor samples were reviewed by one breast pathologist to evaluate the percentage of tumor cells and necrotic areas. If fewer than 60% tumor cells or >40% necrotic area were present on inspection, regions of interest were circled on the H&E-stained slides. The corresponding areas from unstained FFPE tissue sections were then manually macro-dissected for tumor enrichment.

All the clinicopathological data were extracted from electronic medical records (EMR). The overall survival (OS) was defined as the time from diagnosis until death due to any cause, whereas progression-free survival (PFS) was defined as the time from treatment onset until radiological tumor progression, death, or loss to follow-up.

### 2.2. Nucleic Acid Extraction

Total RNA and DNA were isolated from FFPE tissue sections using the AllPrep DNA/RNA FFPE Kit by Qiagen (Hilden, Germany). Paraffin sections were placed in sterile 1.5 mL microcentrifuge tubes, deparaffinized with 100% xylene, and washed twice in 100% ethanol. Deparaffinized tissue was digested with proteinase K at 56 °C for 15 min and then incubated at 80 °C for another 15 min to partially reverse the nucleic acids’ crosslinking. For RNA purification, the samples were DNase-treated and eluted in 30 μL of RNase-free water. For DNA purification, the samples were RNase-treated and eluted in a 30 µL ATE buffer. The total concentration of both DNA and RNA were spectrophotometrically determined using total absorbance at 260 nM, and the purity was quantified using the A260/A280 ratio. RNA samples with A260/A280 ratios of 1.9 ± 0.2 were included in this study.

### 2.3. Gene Expression Profiling

The transcriptional profiles were analyzed using the Affymetrix GeneChip™ Human Gene 2.0 ST Array (Santa Clara, CA, USA), following the manufacturer’s instructions. Briefly, ~200 ng of total RNA was converted into complementary (c)DNA, labeled with the SensationPlus™ FFPE Amplification and WT Labeling^®^ kit (Affymetrix, Santa Clara, CA, USA) and hybridized on the array, which detects both mRNA and lncRNA. The arrays were washed, stained, and scanned using a GeneChip Scanner 3000 7G (Affymetrix, Santa Clara, CA, USA) [25]. The raw data were background-corrected using Robust Multiarray Average (RMA) [26] and normalized with quantile normalization. Differential expression was determined using linear statistical models with arbitrary coefficients, and one contrast of interest was analyzed using the Bioconductor library limma [27]. Correction for multiple hypotheses was applied using false discovery rate (FDR) [28]. Genes were selected based on an absolute value for the fold-change |FC| > 2 and a *p*-value < 0.0002. The raw and normalized data are available at the gene expression omnibus (GEO) repository with accession number GSE176128. Gene expression data were used to classify the samples according to the TNBC-type algorithm and to analyze for differential expression. A gene set enrichment analysis was performed using MetaCore Clarivate™ version 2021 (Clarivate Analytics, Paris, Francia).

### 2.4. Classification of TNBC Patients in IM and Non-IM Subtypes

The web-based TNBCtype algorithm (http://cbc.mc.vanderbilt.edu/tnbc/, accessed on 5 June 2021 was used to identify the TNBC mRNA-based subtypes. Based on previously identified centroids, the method establishes six different signatures for subtypes within TNBC: two basal-like (BL1 and BL2), immunomodulatory (IM), mesenchymal (M), mesenchymal stem-like (MSL), and luminal androgen receptor (LAR). All 68 samples were inserted into the algorithm, which classified 55 samples into one of the aforementioned subtypes; in total, 13 samples were labeled as unclassified. This subset of 55 samples was further grouped into the IM subtype (*n* = 16) and the non-IM subtype (*n* = 39) and used for differential expression analysis on coding and non-coding regions of the transcriptome. Within the non-IM group, sample classification included the BL1 & BL2 (*n* = 20), M (*n* = 12), MSL (*n* = 1), and LAR (*n* = 6) subtypes (Figure S1).

### 2.5. Immunohistochemistry of Tumor Sections

TNBC is diagnosed based on immunohistochemistry (IHC); the antibodies used were: ER (clone 1D5, Dako, Carpinteria, CA, USA), PR (clone PgR636, Dako, Glostrup, Denmark), HER2 (K5204, Dako, Glostrup, Denmark), and Ki67 (clone Clone MIB-1, Dako, Glostrup, Denmark). Histopathologic analysis of strml and intT lymphocytic infiltration was performed on full-face hematoxylin- and eosin (HE)-stained sections. The intT-TILs are defined as lymphocytes in direct cell-to-cell contact with tumor cells with no intervening stroma, while strml-TILs are scattered or clustered between the carcinoma cells/clusters in the stroma and do not directly interact with tumor cells. Tissue microarrays (TMA) of 5 mm were cut into 2 µm sections with the use of a rotary microtome (RM2125 RTS, Leica Biosystems, Nussloch, Germany) and placed on Matsunami TOMO IHC adhesive glass slides (Ventana, Durham, NC, USA). FFPE sections (2 μm) from the TNBC samples were deparaffinized with xylene and rehydrated with a graded ethanol series (100%, 95%, 70%) to distilled water according to standard immunohistochemical protocols. The staining specificity for the IHC was determined using a set of tumor tissues processed in whole sections, with the same fixation and processing methods as the TNBC samples. The optimal concentration of each antibody was established by performing serial titrations on serial FFPE sections. Antigen retrieval conditions and detection methods were also optimized for each antibody to improve sensitivity and signal-to-noise ratio. Briefly, heat-induced antigen retrieval was performed by placing the slides in a Tris-EDTA (pH 9) or citrate (pH 6) buffer for 20 min at 98 °C using a water bath. The tissue sections were cooled in the buffer for 20 min before the Peroxidase Blocking Reagent (Dako) treatment for 10 min. The slides were then incubated with Background Sniper (Biocare, Pacheco, CA, USA) for 20 min, and then with anti-CD4 (1:30, clone BC/IF6, Dako, Glostrup, Denmark), anti-CD8 (1:50, clone SP16, Dako, Glostrup, Denmark), anti-FOXP3 (1:100, clone 86D, Biocare, Pacheco, CA, USA), anti-PD-1 (1:200, clone NAT105, Abcam, Cambridge, Cambridgeshire, UK), anti-PD-L1 (1:20, clone 28-8, Abcam, Cambridge, Cambridgeshire, UK), and anti-CTLA-4 (1:20, clone F-8, Santa Cruz Biotecnology, Santa Cruz, CA, USA) primary monoclonal antibodies. After washing in PBS, DAKO Envision systems (Dako) or MACH 1 Universal HRP Polymer (BioCare) and diaminobenzidine (DAB; BioCare) were used for chromogenic immunodetection, followed by counterstaining with hematoxylin. Negative control slides without primary antibodies and positive controls for each marker were used for each immunostaining run. Briefly, each section was reviewed at low magnification. Positive lymphocytes in tumor stroma were counted in three high-power fields (HPF; ×40; Olympus BX53, Life Science Solutions, Chicago, IL, USA), representing the staining spectrum seen on the whole section’s initial overview and displayed as the average number of stained cells per HPF. A breast cancer expert pathologist, blinded for patient characteristics and outcome, evaluated the TILs and performed the IHC analyses.

### 2.6. Next-Generation Sequencing

Genetic libraries for sequencing were generated for each DNA sample with the commercial capture-based target enrichment panel Solid Tumor Solution STS_v1 (Sophia Genetics, Saint Sulpice, Switzerland). The sequencing was performed using an Illumina MiniSeq instrument (Illumina, Foster City, CA, USA). Multiplexed runs, including 15 libraries each, were carried out using 300 cycle MiniSeq High Output Kits (Illumina, San Diego, CA, USA). The fastq files were analyzed for their quality with FastQC [29] and filtered with trimmomatic [30] before being aligned to the reference genome (GRCh38). The targeted coding exons and splice junctions of known protein-coding RefSeq annotated genes were assessed for an average depth of coverage, with a minimum depth of 30× required for inclusion in downstream analysis. Local realignment around insertion-deletion sites and regions with poor mapping quality was performed using GATK HaplotypeCaller (Broad Institute, Cambridge, MA, USA) [31], conducting a base quality score recalibration. Variant calls were also identified using GATK HaplotypeCaller. Variants were filtered out based on inheritance patterns, variant type, gene panel, phenotype, and population frequencies. The SOPHiA DDM platform (Sophia Genetics, Lausanne, Switzerland) panel comprehensively assesses target regions in 42 cancer-associated genes (Table S1). The resources included are the HGMD, 1000 Genomes database, RefSeq Genes 109.20201120 v2, NCBI, Transcript Interactions RefSeq Genes 109.20201120 v2, NCBI, gnomAD Exomes Variant Frequencies 2.0.1, Broad Institute of Harvard & MIT NHLBI GO Exome Sequencing Project, OMIM, PubMed, ClinVar and GenVisR [32].

### 2.7. Statistical Analyses

The summary statistics, including means, medians, ranges, and standard deviations, were calculated for continuous variables; the categorical variables were summarized as proportions and confidence intervals (95% CIs). Significant differences among continuous variables for non-parametric distributions were assessed using the Mann–Whitney U test. The Chi-square test or Fisher’s exact test was used to determine statistically significant differences among categorical variables. The PFS and OS were analyzed using the Kaplan–Meier method, while the log-rank test evaluated the differences between subgroups. Univariable and multivariable analyses of the PFS and OS were performed using the Cox proportional hazards model. In addition, because death may preclude observation of progression, we conducted competing risks analyses with progression as the event of interest and death as the competing event. Cumulative incidence functions were estimated for each subgroup and compared using Gray’s test, and subdistribution hazard ratios (SHRs) with 95% CIs were derived from Fine–Gray regression (univariable and multivariable), adhering to the same two-sided significance threshold (*p* < 0.05). Statistical significance was predetermined at a *p*-value < 0.05 based on a two-sided test. The analyses were performed using SPSS version 26 (IBM Company, Armonk, NY, USA) and R statistical software version 4.1.1 (R Core Team, Vienna, Austria). Competing risks analyses were performed in R (package cmprsk).

## 3. Results

### 3.1. Clinicopathological Features of TNBC Patients

A total of 68 TNBC patients were enrolled in this study, with a median age of 49.5 (30–80) years at diagnosis and an equal proportion of pre- and post-menopausal samples. The most frequent clinical classification at diagnosis was stage III (83.8%; *n* = 57), based on the American Joint Committee on Cancer (AJCC) Staging Manual (8th edition). At least one high-risk clinicopathological characteristic was reported for all the clinical stage III patients. High-grade tumors were identified in 88% (*n* = 60) of the samples. Ductal histology was present in 87% of patients. Vascular infiltration was observed in 47% (*n* = 32), and more than half of the cohort (55.9%; *n* = 38) featured a high level (>20%) of Ki67 (Table 1).

### 3.2. Treatment Management of TNBC Patients

All the patients with locally advanced disease (83.8%; *n* = 57) received neoadjuvant chemotherapy; of these, only 14 (24.6%) underwent a pCR, and 43 (75.4%) underwent a radical modified mastectomy. The preferred neoadjuvant treatment was an anthracycline and taxane-based chemotherapy regimen; notably, 42 (61.8%) patients received platinum as a third agent. For patients with clinical stage IV, anthracycline- or taxane-based chemotherapy was administered. Remarkably, immunotherapy was not administered, neither in the locally advanced nor in the metastatic setting, mainly due to access barriers.

Out of the cohort of 57 patients with clinical stage III disease, 40 (70.2%) had at least one high-risk pathological feature (positive axillary lymph nodes, histologic grade 3, Ki-67 index > 20% or lymphovascular invasion). Forty patients had at least one high-risk pathological feature (positive axillary lymph nodes, histologic grade 3, Ki-67 index >20% or lymphovascular invasion), among which the patterns of recurrence were the following: 25 (43.9%) had central nervous system (CNS) involvement, 21 (36.8%) lung metastases, 12 (21.1%) bone metastases, and 11 (19.3%) developed liver metastases (Table S2).

### 3.3. Classification of TNBC Patients into IM and Non-IM Subtypes

According to the Lehmann subtypes, out of the 68 tumor samples, 55 were classified as IM and non-IM (Table 2). The IM subtype was the most common in our TNBC cohort, being found in 16/68 (23.5%) of the cancers examined, followed by the BL1 and BL2 subtypes found in 20% of cases. Figure S1 shows the complete analysis per sample based on Lehmann subtypes. The Strobe flow diagram in the Supplementary Materials describes further details regarding the complete analysis of tumor samples (Figure S2).

### 3.4. TILs Subpopulations in the IM Subtype

**Figure 1:**
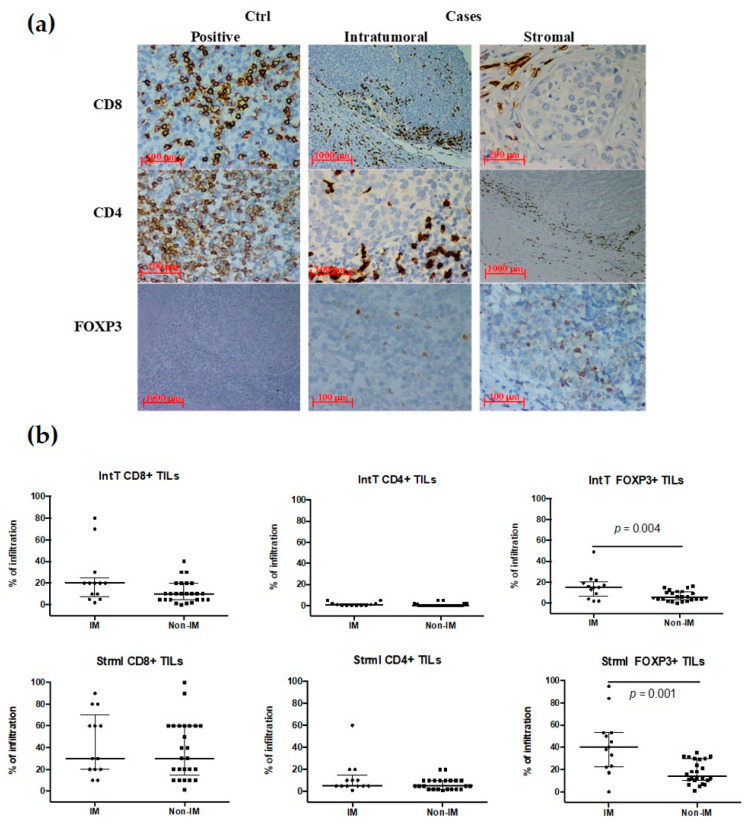
Tumor infiltration lymphocytes (TILs) in the immunomodulatory (IM) subtype vs. non-IM subtype. (**a**) Representative images of immunohistochemistry depicting both intratumoral (intT) and stromal (strml) infiltrating lymphocyte subpopulations (CD4+ (ctrl ×40, intT ×40, strml ×4); CD8+ (ctrl ×40, intT ×4, strml ×20), and FOXP3+ (ctrl ×4, intT ×40, strml ×40)) in TNBC biopsies. (**b**) Scatter plots (percentages) compare median and data distribution between IM vs. non-IM subtypes, both intT and strml per lymphocytes subpopulation (with interquartile range bars and the median); differences assessed using the Mann–Whitney *U* test; statistically significant differences are shown in each plot. Ctrl: controls.

Thirty-nine specimens were available for the TILs subpopulation analysis: IM subtype (*n* = 13) and non-IM subtypes (*n* = 26). The analysis showed a higher but non-significant intT infiltration of CD8+ (Th1) TILs in the IM subtype when compared with the non-IM subtype (20% (95% CI: 9.4–38.6) vs. 10% (7.16–15.45); *p* = 0.058) (Figure 1). Moreover, no significant differences were identified between the two subtypes in terms of CD8+ strml TILs. For CD4+ TILs (Th2), we did not identify any associations regarding the intT nor strml infiltration and the tumor subtypes.

Lastly, the IHC analyses showed that the most predominant TILs phenotypes in the TNBC samples were Treg cells (FOXP3+). Interestingly, most cells featured positive cytoplasmic staining vs. nuclear for this particular marker. A significant difference was observed when comparing the proportion of FOXP3+ intT cells in IM vs. non-IM subtype tumors (15% vs. 5%;95%CI, 8.49–23.21; *p* = 0.004). In the case of strml this difference was also statistically significant between the IM and non-IM subtypes (40% vs. 14%; 95%CI, 26.80–58.27; *p* = 0.001) (Figure 1).

### 3.5. Differential Expression of Coding and Long Non-Coding RNAs

**Figure 2:**
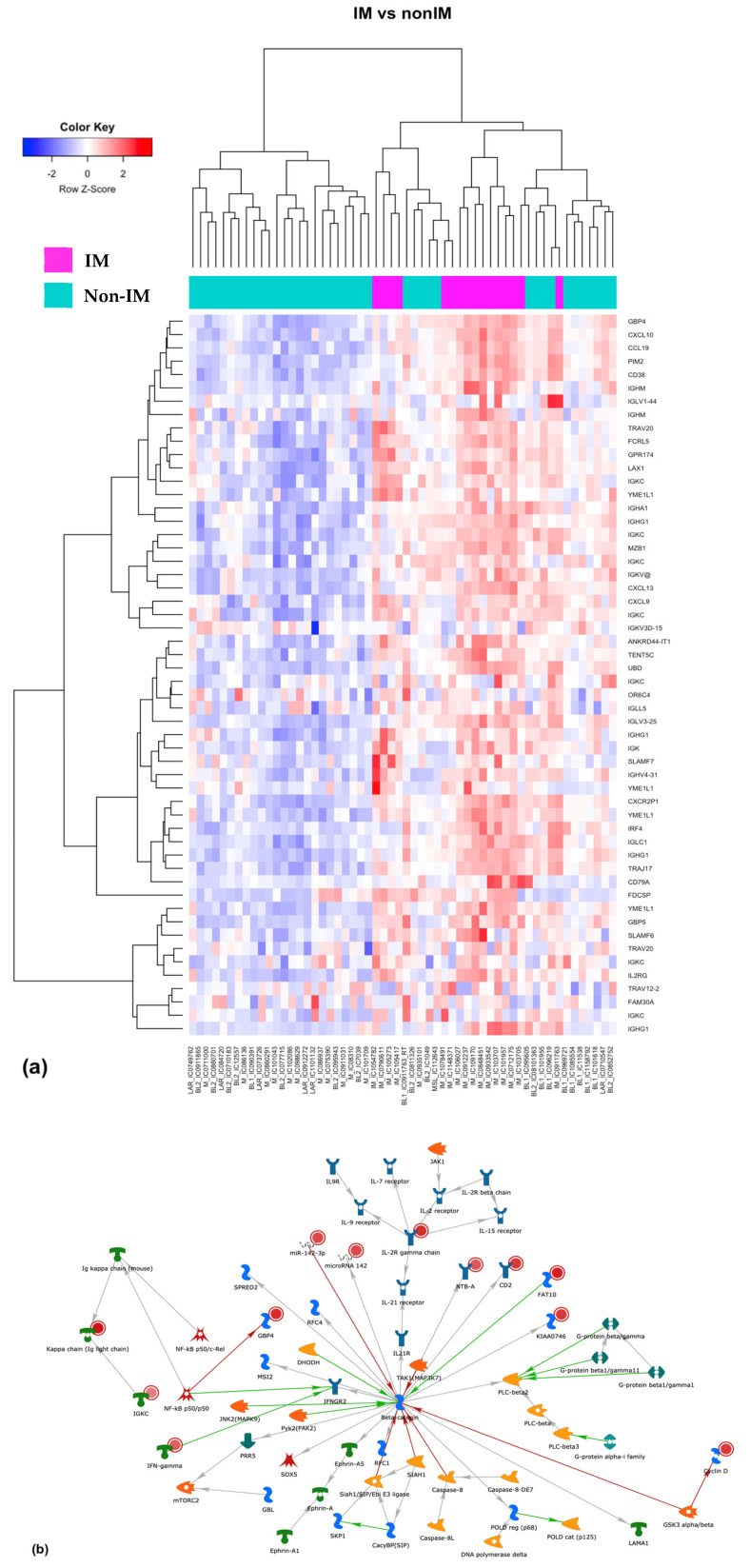
Gene expression analysis (**a**) heat map resulting from an unsupervised hierarchical clustering, the top bar of the heat map shows in magenta the IM samples and in light green the non-IM. Red indicates gene overexpression; columns are samples and rows are genes. (**b**) Enrichment analysis using Metacore Clarivate™ highlighted the β-catenin signaling pathway; red dots mark the genes in our list.

We conducted a differential gene expression analysis on both coding and long non-coding regions in order to characterize differences among samples classified as IM and non-IM subtypes. The results from this analysis showed a total of 74 genes with differential expression (DEGs) when comparing IM vs. non-IM samples. In all cases, differential expression resulted in a significant up-regulation of these genes in the IM subtype, with |FC| > 2 and a *p*-value < 0.0002 (Table S3). We further performed unsupervised hierarchical clustering (Figure 2a), which identified several patterns in terms of DEGs. This is illustrated in the heatmap shown in Figure 2a, in which the IM samples (shown in magenta) clustered to show a pattern indicating gene overexpression, compared with non-IM samples (shown in teal). To explore gene function, a gene set enrichment analysis of the genes expressed in the IM group identified biological processes associated with the immune system relevant to our working hypothesis. Pathway analysis revealed enrichment of the β-catenin signaling pathway (known to be highly relevant in human cancers) (Figure 2b) in 16% of the genes we identified as differentially expressed.

### 3.6. LncRNAs Associated with the IM Subtype

The differential expression analysis of the long non-coding regions of the transcriptome between the IM and non-IM samples was sparser compared with the coding sequences. From this analysis we identified seven long non-coding RNAs differentially expressed (six up-regulated (↑) and one down-regulated (↓)) based on |FC| > 2 and a *p*-value < 0.006 (LINC00173↑, LINC00854↑, LINC00869↑, LINC00426↑, LINC00861↑, LINC01550↑, LINC00312↓) (Table 3).

**Figure 3:**
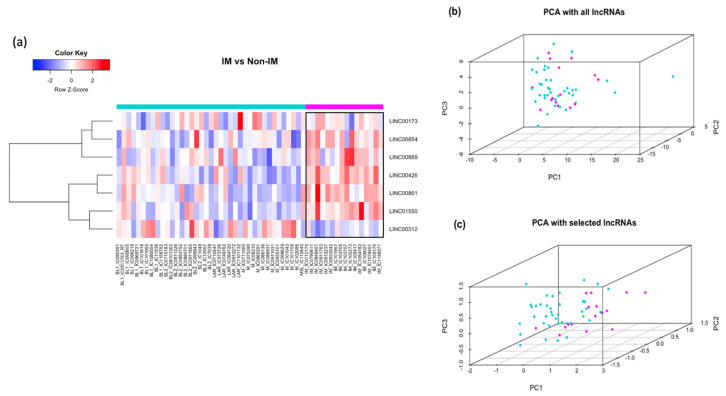
Differential expression of lncRNA between IM vs. non-IM subtypes. (**a**) Heat map from an unsupervised hierarchical clustering of selected lncRNAs based on fold-change ≥ 1.2 and significance level *p*-value < 0.006. The top bar in magenta shows samples classified as IM; the teal bar represents those classified as non-IM. Red represents up-regulation and blue down-regulation. (**b**) Principal component analysis (PCA) showing a 3D plot of IM (magenta) vs. non-IM (teal) samples when all annotated lncRNAs are used. (**c**) The same plot when using only the seven most significant lncRNAs, which better classifies the IM and non-IM subtypes under analysis. PC: principal components; lncRNA: long non-coding RNA, IM: immunomodulatory.

Figure 3a shows the results from the unsupervised hierarchical clustering approach, highlighting the patterns of expression clustered by subtype. The figure shows an opposite expression pattern among the mesenchymal subtype and the IM subtype samples.

A principal component analysis (PCA) was performed to evaluate the effect of those seven long intergenic non-coding RNAs separating the IM from the rest of the subtypes. Figure 3b displays the PCA using all annotated lncRNAs in the microarray where the first three principal components can only accumulate 31.7% of the total variability in the data for all the samples. By contrast, Figure 3c shows the PCA using only the seven statistically significant lncRNAs; in this case, the first three principal components account for 77.43% of the total variability. Though the classification is not perfect, we were able to identify those that best separate the two groups. Summary statistics after fitting the linear model are presented in Table 3. All seven lncRNAs were statistically significant according to the *p*-value, but only five remained significant after FDR adjustment for multiple hypotheses represented in the adjusted *p*-value.

### 3.7. PD-1, PD-L1, and CTL-4 Expression in the IM Subtype

**Figure 4:**
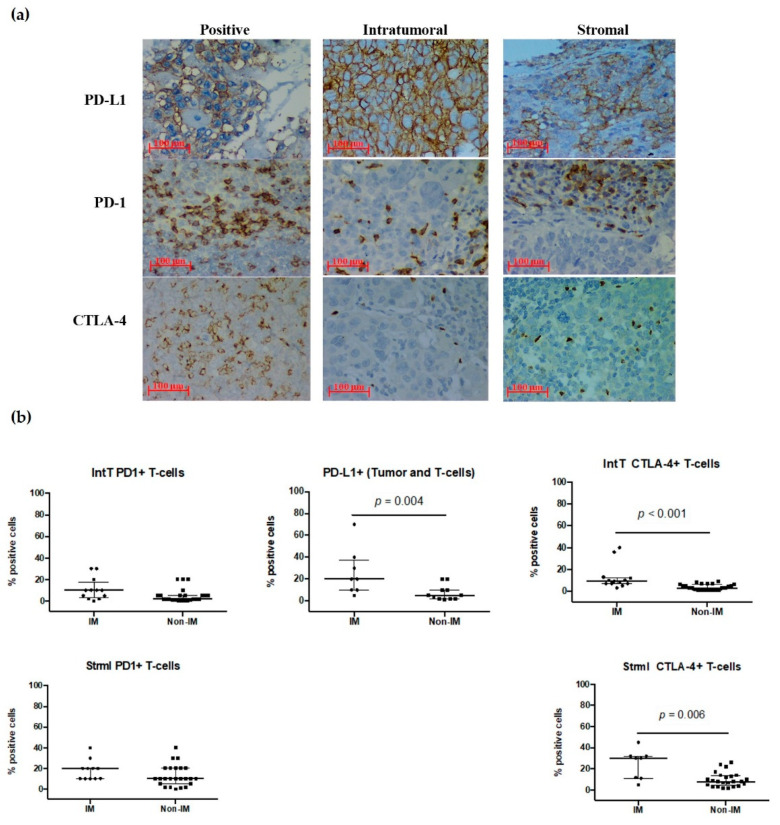
Expression of immune checkpoints in IM vs. non-IM subtypes. (**a**) Immunohistochemistry images of both intratumoral (intT) and stromal (strml) immune checkpoints (ICp) [PD-L1+ (ctrl ×40, intT ×40, strml ×40); PD-1+ (ctrl ×40, intT ×40, strml ×40), and CTLA-4+ (ctrl ×40, intT ×40, strml ×40)] in TNBC biopsies. (**b**) Scatter plots (percentages) comparing median and data distribution between IM vs. non-IM subtypes, both intT and strml per ICp positive cells (with interquartile range bars and the median); differences assessed using the Mann–Whitney *U* test; statistically significant differences are shown in each plot. Ctrl: controls.

Even though PD-1+ TILs were identified in 97.4% of the tumor samples in the IM subtype group, PD-1+ expression in tumor cells did not differ significantly between each subtype. In the other assessments, we identified a clear increase in expression for the IM subtype compared with the non-IM (Figure 4a). In the case of PD-1+ TILs, we observed an increase in the IM subtype for both intT 10%(IM) vs. 2%(non-IM) (*p* = 0.051), and strml 20%(IM) vs. 10%(non-IM) (*p* = 0.095), although this difference did not reach statistical significance (Figure 4b). However, PD-L1 + expression in tumor cells was significantly higher (*p* = 0.004); CTLA-4+ TILs were also significantly higher in intT (*p* < 0.001) and strml (*p* = 0.006) assessments, respectively.

### 3.8. Comparison of Mutation Frequency between IM and Non-IM Subtypes

**Figure 5:**
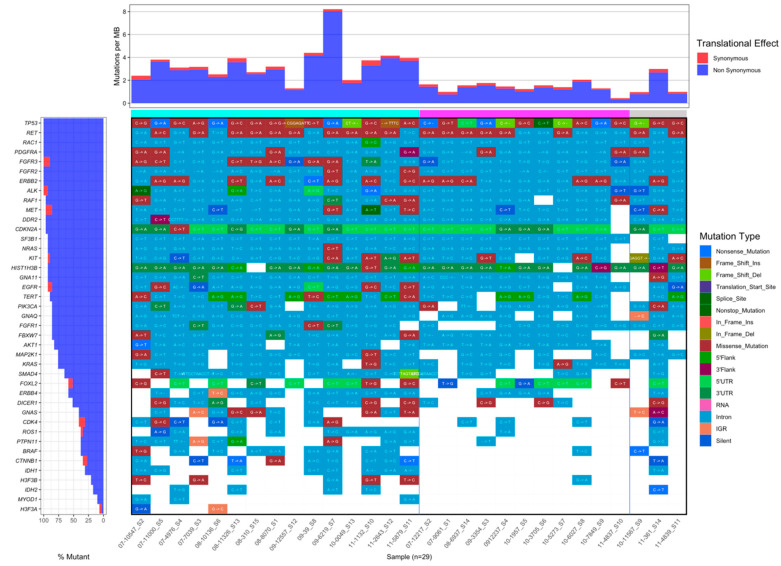
Comparison of pathogenic mutation by type and frequency in IM subtype (magenta) and non-IM subtypes (teal) according to the Lehmann classification for TNBC patients. The last three samples (light gray) are unclassified samples, which were sequenced for comparison reasons. The top bar shows the mutation rate by translational effect per sample, and the left bar plot shows the mutation rate and its translational effect by gene; synonymous variants are marked in red and non-synonymous in blue. The top mutated gene is *TP53*, followed by *RET*, *RAC1*, and *PDGFRA*.

We identified genetic alterations in 42 clinically relevant genes associated with solid tumors in 29 TNBC samples (11 IM and 18 non-IM) (Figure 5). A total of 3216 somatically acquired base substitutions were identified; among these, 2294 were in intronic sites. There were 162 missense, 13 nonsense, 3 essential splice-site, and 281 silent mutations in the protein-coding regions. A total of 176 intergenic region mutations were observed in 40% of genes included in this 42 gene panel. Out of the nine indels identified, five were frame-shift deletions, four in *TP53* and one in *SMAD4*; two in-frame deletions were identified, one in *TP53* and the other in *KIT*; additionally, two insertions, both in *TP53,* were observed. The summary of the mutations is shown in Figure 5.

We further observed a difference in terms of the mutation rate between the IM (top bar in magenta) and non-IM (top bar in teal) subtypes. Within the latter, there were three samples that could not be classified using Lehmann’s algorithm (far-right columns with the top bar in light grey). The blue/red bars on top of the main plot show the mutation rate per sample, represented as synonymous (red) and non-synonymous (blue) alterations. Notably, the non-IM subgroup is characterized by a higher number of mutations, and a higher abundance of missense variants. At the bottom of the left bar plot, low-mutated genes can be observed, featuring a sparse mutation pattern for the IM samples. We found five mutation-free genes (*GNAS*, *CTNNB1*, *IDH1*, *IDH2*, *MYOD1*) and two that might be potential biomarkers for TNBC immunotherapy; *CTNNB1* (Catenin beta-1) and *IDH1*. For IDH, no mutations were found for the IM samples and 50% of the non-IM samples featured a mutated version of this gene. For the case of *IDH2*, no mutations were found in the samples from the IM group and 28% in the non-IM samples; all but one was intronic, and the other was a non-sense mutation.

### 3.9. Clinical Variables and Outcomes

**Figure 6:**
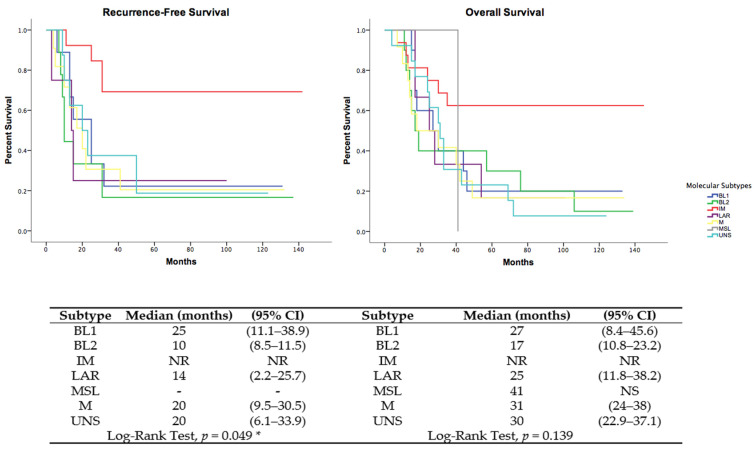
Kaplan–Meier curves for recurrence-free survival in patients with stage III disease (**left**) and overall survival (**right**) for all samples classified with different molecular TNBC subtypes. * *p* < 0.05. CI: confidence interval; BL1 basal like 1; BL2: basal like 2; IM: immunomodulatory; MSL: mesenchymal stem-like; M: mesenchymal; NR: not reached; NS: non-significant.

The clinical and pathological variables associated with the IM subtype included vascular invasion and low probability of metastasis to the lung, liver, and central nervous system (Table S4). The Kaplan–Meier curves for recurrence-free survival (RFS) in stage III patients and for overall survival are shown in Figure 6. A total of 51 (75%) patients died due to disease progression during follow-up. In both plots, we observe how different the survival rate is for IM subtype patients compared to each of the other subtypes. This result suggests that the IM subtype featured the more favorable prognosis of all molecular subtypes.

In the multivariate analysis, the type of breast surgery and previous CNS radiotherapy increased the risk of death and recurrence significantly. Interestingly, after adjusting for significant factors in the univariate analysis, the IM subtype positively influenced RFS and OS in patients with TNBC, representing a protective factor. The complete model is shown in Table S5. Using the pooled M and UNS tumors as the reference, the immunomodulatory (IM) subtype showed an sHR of 0.25 (95 % CI 0.08–0.81, *p* = 0.021). All other subtypes mirrored the non-significant behavior reported in the KM and multivariable Cox models (Table 4).

## 4. Discussion

Currently, immunotherapy benefits seem to be confined to TNBC PDL-1 positive patients in advanced stages or earlier stages as a neoadjuvant strategy [16,19,33]. Remarkably, contradictory data [34] have undermined currently approved immunotherapy indications [35], emphasizing the necessity of continuing to explore and characterize the TNBC subtype. Even though some immune subclassifications have been developed, to date, none has been incorporated systematically into daily clinical practice. Nonetheless, individualized therapies have been suggested according to each TNBC subtype, based on molecular and immune data [36].

The present study presents relevant information derived from a cohort of 68 TNBC patients classified by the Lehmann algorithm (*n* = 55), who were grouped according to immunomodulation into IM (*n* = 16) and non-IM (*n* = 39) subtypes. Lehmann et al. excluded the IM and MSL subtype in the more recent TNBC classification, arguing that the transcripts in the original classification came from lymphocyte infiltration and tumor-associated stromal cells infiltration of immune cells rather than the tumor cells. The recent advent of immunotherapy in different clinical scenarios of the TNBC and the evidence from translational medicine studies have supported the necessity of visualizing this disease from a global point of view and pursuing the characterization of the immune milieu and the whole tumor microenvironment instead of a reductionist approach limited to characterizing just the tumor. [37,38,39]. We present a multi-omics analysis complemented with clinical data contrasting the behavior of IM vs. non-IM disease subtypes, exploring protein-coding genes, long intergenic non-coding RNAs, TILs, DNA mutation rates, clinical, histopathological, and survival patterns.

Gene expression analysis on coding genes identified a list of 74 DEGs, all up-regulated in IM when contrasted with the non-IM samples. The gene set enrichment analysis revealed that the β-catenin pathway is associated with 16% of the genes differentially regulated in the IM subtype in our study. It has been reported that the Wnt/β-catenin signaling is associated with cancer; this signaling disrupts the tumor-immunity in most nodes, including T cells and tumor cells cycle, facilitating cancer development and preventing spontaneous T-cells infiltration across most human cancers [40,41]. Both the canonical and non-canonical Wnt/β-catenin pathways are essential for mammary gland development [42] and BC growth and dissemination [43]. In fact, breast cancer patients with activation of the WNT/ β-catenin pathway have been associated with earlier stages of the basal-like subtypes, suggesting that the activation of this pathway might be an early event in breast cancer genesis [44]. A recent study showed that the β-catenin inhibitor enhanced T-cell infiltration, shifting the tumor microenvironment into a T-cell-inflamed phenotype. Furthermore, it has been reported that β-catenin status could potentially serve as a clinical predictor of immunotherapy outcome [45]. Therefore, we propose that the Wnt/β-catenin pathway might be evaluated in future studies exploring the treatment effect in combination with immunotherapy [46].

On the other hand, accumulating evidence indicates that lncRNAs are encouraging biomarkers involved in regulating gene expression and cancer biology [47]. One example is lncKLHDC7B, which was involved as a transcriptional modulator of proapoptotic signals and avoided cell migration and invasiveness [48]. According to Lehmann’s classification, we explored these non-protein-coding molecules through a differential expression analysis between the IM subtype and non-IM subtypes. The analysis identified seven lincRNAs associated with the TNBC-IM subtype; this subtype showed a better prognosis compared to non-IM subtypes. Our study demonstrated an opposite expression pattern of lncRNA expression between the IM subtype and mesenchymal type, both reported in immune regulation. In this regard, MSL cells promote inflammation when the immune system is not fully activated or limit inflammation if the immune system is overwhelmed as it fights cancer [49]. Of those seven lincRNAs, LINC01550 and LINC00426 were identified as oncogenesis regulators in non-BC tumors [50,51] and only three (LINC00173, LINC00861, LINC00312) were identified in BC [52,53,54]. However, we found evidence of association in four regarding tumor immune regulation: LINC00426, LINC00173, LINC00861, and LINC00312. LINC00426 has been associated with an increase in CD8+ TILs and macrophage M2 infiltration [55]. Two more, LINC00173 [56] and LINC00861, regulate immune signaling pathways and immune checkpoints, specifically PD-1 and CTLA-4 [57]; and as we discuss next, CTLA-4 was found with high expression in the IM subtype. LINC00312 overexpression has been reported as an inducer of aggressiveness in tumors [58] and was found to be downregulated in the IM subtype in our study, from which we could associate this downregulation to a good prognosis. Moreover, LINC00312 is associated with the Wnt-β-catenin pathway [59], which was enriched in the IM subtype from the coding genes analysis, and LINC00173 has also been reported as a regulator of β-catenin expression [60]. Furthermore, LINC00861 [61] and LINC00312 [62] are associated with the PI3K/AKT pathway, which is, in turn, associated with immune response [21]. We also found two new lincRNAs upregulated in the TNBC-IM subtype LINC00854 and LINC00869, with little to no association with disease or molecular function. However, altogether, this suggests an involvement in the tumor immune response, and thereby, further studies are required to clarify their signaling pathway involvement.

Mutation rates between the IM and the non-IM subtypes also showed substantial differences. We highlighted two genes because of their relevance to immune system response and because no mutations were found in the IM samples. We presented CTNNB1 (Catenin β-1), a key downstream component of the canonical Wnt signaling pathway [63,64,65,66,67,68,69,70]; it was also found relevant to explain results from the transcriptomic analysis and to discuss the tumor immune-microenvironment regulation. Other pathways are those related to IDH1, the abnormal conversion of 2-oxoglutarate to 2-hydroxyglutarate, and the innate immune system. Although this type of mutation can either make a protein more effective or defective, in this case, it could be the latter; it was notable that the non-IM subtypes demonstrated a worse prognosis than the IM subtype considering only genes relevant in cancer. Further studies are needed to clarify the participation of these genes in the immune milieu.

With the primary aim to ameliorate immunotherapy advances for TNBC, TILs have been associated with better prognosis and predictive value [8,9,12]. However, it is still unclear which immune cell types contribute the most to outcome improvement. This study analyzed TIL subpopulations in the IM subtype of our cohort. The results showed higher FOXP3-TILs (Treg) infiltration in both intT and strml within this subtype. We also observed a trend toward high CD8-TILs (Th1) infiltration. Tumor infiltration, predominantly by Tregs, was associated previously with unfavorable histologic features and poor survival outcomes in breast cancer [71], mainly through transforming growth factor (TGF-β) and IL-2 stimuli promoting an immunosuppressive microenvironment [72].

By contrast, other studies have postulated that an increased number of intT Tregs has been associated with better clinical outcomes, reinforcing the idea that the type of immune cell and interaction is essential, as well as the density and specific location [73]. In line with this, we also found that Tregs may be a favorable prognostic factor for recurrence-free and overall survival in the IM subtype in an independent way to current biomarkers. This has been confirmed mainly when intT Tregs are accompanied by CD8-TILs and CD20+ B cells [74]. Furthermore, Tregs work in conjunction with Th17 cells through TGF-β, thereby prompting differentiation of the Th17 phenotype from CD4+ Th2 cells (in the presence of IL-6) and Tregs [75]. Th17 cells can acquire Th1-like characteristics due to their plasticity (ex-Th17 cells or non-classical Th1 cells) after activation, enhancing the antitumor immunity through pro-inflammatory signals [76]. Therefore, we suggest the key participation of Th17 response in the IM subtype due to low infiltrations of classical Th1 response.

We also report that the IM subtype was characterized by a high CTLA-4 expression of intT and strml T-cells in the IHC analysis, associated with a high PDL-1 positivity in the tumor and increased PD1+ TILs density. Although the clinical significance of ICp is still under debate, PD-L1 expression has been independently associated with a worse prognosis [77]. Similarly, Ren et al. stated that PD-1 was linked with PDL-1 expression and increased density of TILs, which may positively impact and modify prognosis [77]. In the present study, the IM subtype exhibited a longer OS and a more prolonged RFS considering just high-risk stage III patients. However, the lack of standardization in the staining procedures of ICps in TNBC may result in inconsistent results and performance in clinical studies.

PD-L1 positivity has been reported from 19% to 64% [78]; herein, the IM tumor samples showed 20% PD-L1 positivity, 30% intT CTLA-4, and 9% strml CTLA-4 expression, which are in agreement with previous reports. Leisha et al. reported a sub-analysis of IMPASSION 130 following three distinct immune subtypes: inflamed, immune exclude, and desert; the inflamed subtype presented the highest PD-L1 expression. Another key finding was its benefit from immunotherapy (HR 0.63, 95% CI 0.49–0.81) [42], which is encouraging and influenced this study [79].

Adverse and immune-related adverse events associated with immunotherapy administration are more prevalent with CTLA-4 inhibitors [80]. Th17 cells and IL-17 might play an essential role in the efficacy and toxicity of ICIs, wherein toxicities have correlated with antitumor response [81]. The immunoregulatory role of Th17 and its influence on toxicity could be evaluated in the future to increase tolerability and adherence to treatment. Considering the high positivity of CTLA-4 staining in the IM subtype and the potential benefit of this immune pathway may be promising to assess its efficacy with an optimal treatment administration.

Our study features some limitations. We recognize that the small sample size and retrospective nature did not include patients treated with immunotherapy. However, the hypotheses did not include before and after immunotherapy but, rather, molecular and clinical differences that support the TNBC subtype classification by Lehmann. The sample size also precludes the performance of a subset analysis of TILs and their association level, as well as immune signature scores with clinical characteristics, which were carefully conducted. TMA may incorporate some bias; however, we worked around this by sampling three representative regions from every tumor.

## 5. Conclusions

TNBC is a highly aggressive breast cancer subtype. This study presents evidence regarding the IM subtype within TNBC. Our results show that it features an increased FOXP3+ TILs infiltration and a high CTLA-4 and PD-L1 immune checkpoints expression. We also identified long intergenic non-coding RNAs associated with immune responses. The transcriptomic and enrichment analyses identified the β-catenin signaling pathway characterizing the IM subtype from other breast tumor microenvironments. The DNA sequencing analysis revealed differences in both mutation rates and mutation type. Overall, we were able to identify novel lincRNA biomarkers from a thorough characterization of the molecular and immune milieu of the IM subtype, highlighting the importance of algorithms for patient classification but also for the design of future clinical trials in which an immune-based therapy could benefit this subgroup of patients.

## Figures and Tables

**Table 1:** Baseline Clinical and Pathological Characteristics of Patients (*n* = 68).

Variables	% *n*/*n*
Age (years) median (min–max)	49.5 (30–80)
≥40	85.3 (58/68)
<40	14.7 (10/68)
Hormonal Status	
Premenopausal	50 (34/68)
Postmenopausal	50 (34/68)
Clinical Stage	
III	83.8 (57/68)
IV	16.2 (11/68)
Surgical procedure (mastectomy)	
Yes	75.4 (43/57)
No	24.6 (14/57)
Histology	
Ductal	86.8 (59/68)
Lobular	7.4 (5/68)
Other	5.9 (4/68)
Nuclear Grade	
G3	88.2 (60/68)
G2	11.8 (8/68)
Vascular Infiltration	
Yes	47.1 (32/68)
No	52.9 (36/68)
Pathological Complete Response	
Yes	24.6 (14/57)
No	50.9 (29/57)
NE	24.6 (14/57)
Systemic Treatment	
Neo/Adjuvant	83.8 (57/68)
Palliative	16.2 (11/68)
Radiotherapy	
No	25 (17/68)
Yes	75 (51/68)
Ki67(%)	
<14	1.5 (1/68)
14–20	2.9 (2/68)
>20	55.9 (38/68)
NE	39.7 (27/68)
CEA (ng/mL)	
<3.38	79.4 (54/68)
>3.38	19.1 (13/68)
NE	1.5 (1/68)
CA 15-3 (U/mL)	
<12.32	20.6 (14/68)
>12.32	77.9 (53/68)
NE	1.5 (1/68)
Chemotherapy Type	
A + T	22.0 (15/68)
A + T + Cis	61.8 (42/68)
A or T	16.2 (11/68)

CAE: carcinoembryonic antigen; CA 15-3: Carbohydrate antigen 15-3; A + T: Adriamycin + taxane; A + T + C: Adriamycin + taxane + cisplatin; NE: Not evaluated.

**Table 2:** Molecular Subtype by Lehmann et al. [4].

IM and Non-IM	Subtype	% (*n*/*n*)
IM	IM	23.5 (16/68)
Non-IM 57.3 (39/68)	BL1	14.7 (10/68)
	BL2	14.7 (10/68)
	LAR	8.8 (6/68)
	M	17.6 (12/68)
	MSL	1.5 (1/68)
	UNS	19.1 (13/68)

IM: Immunomodulatory; BL1: basal like 1; BL2: basal like 2, M: mesenchymal; MSL: mesenchymal stem-like; LAR: luminal androgen receptor; UNS: unclassified.

**Table 3:** Summary statistics from a linear model to determine long non-coding RNAs differential expression in IM vs. non-IM samples.

lncRNA	FCH	Mean Expression	*t*-Test	*p* Value	adj. *p* Value
LINC00861	1.64	4.800199	4.982293	5.82 × 10^−6^	0.00182405
LINC00869	1.49	6.408327	3.61491	6.23 × 10^−4^	0.083770344
LINC00426	1.38	4.920571	5.373881	1.38 × 10^−6^	0.000647671
LINC01550	1.35	4.084667	5.5031	8.50 × 10^−7^	0.000647671
LINC00854	1.32	6.853894	3.699587	4.76 × 10^−4^	0.074699771
LINC00312	−1.29	3.827583	−2.835162	6.26 × 10^−3^	0.317551682
LINC00173	1.26	4.548792	3.099832	2.97 × 10^−3^	0.199410413

LINC: long intergenic non-coding; FCH: fold-change; adj. adjusted.

**Table 4:** Competing risk analysis.

TNBC Molecular Subtype	Sub-Distribution Hazard Ratio (SHR)	Robust s.e.	z-Score	*p*-Value	95% CI (Lower)	95% CI (Upper)
BL1	1.0238	0.5039	0.05	0.962	0.3901	2.6865
BL2	1.3908	0.7935	0.58	0.563	0.4546	4.2549
IM	0.2545	0.1511	−2.31	0.021	0.0795	0.8146
LAR	1.3317	1.0144	0.38	0.707	0.2992	5.9265
MSL	1.047	0.5395	0.09	0.929	0.3813	2.8747
M	1.0000 ^†^	—	—	—	—	—
UNS	1.0000 ^†^	—	—	—	—	—

^†^ Mesenchymal (M) and unclassified (UNS) subtypes served jointly as the reference category and were omitted by the model software; their SHR is therefore fixed at 1.

## Data Availability

The microarray data presented in this study are available on GEO Accession number GSE176128. The sequencing data are available upon request to the corresponding author. The clinical data are not publicly available due to ethical institutional policies regarding patient confidentiality.

## Supplementary Materials

The following are available online at https://www.mdpi.com/article/10.3390/cancers13246256/s1, Figure S1: Distributions of the Vanderbilt subtypes in Mexican women with triple-negative breast cancer (TNBC) (*n* = 68): basal-like 1 (BL1), basal-like 2 (BL2), immunomodulatory (IM), luminal androgen receptor (LAR), mesenchymal (M), and mesenchymal stem-like (MSL), Figure S2: Strobe flow diagram, Table S1: Gene content of cancer panel, Table S2: Type of Recurrence Sites of Stage III Patients (*n* = 57), Table S3: Differential gene expression analysis coding and long non-coding regions; Table S4: Clinical and pathological characteristics of TNBC patients with IM and non-IM subtypes, Table S5: Univariate and multivariate analysis with RFS and OS of TNBC patients.
