# Correction: Rodríguez-Bautista et al. Immune Milieu and Genomic Alterations Set the Triple-Negative Breast Cancer Immunomodulatory Subtype Tumor Behavior. *Cancers* 2021, *13*, 6256

**DOI:** 10.3390/cancers17193212

**Published:** 2025-10-01

**Authors:** Rubén Rodríguez-Bautista, Claudia H. Caro-Sánchez, Paula Cabrera-Galeana, Gerardo J. Alanis-Funes, Everardo Gutierrez-Millán, Santiago Ávila-Ríos, Margarita Matías-Florentino, Gustavo Reyes-Terán, José Díaz-Chávez, Cynthia Villarreal-Garza, Norma Y. Hernández-Pedro, Alette Ortega-Gómez, Luis Lara-Mejía, Claudia Rangel-Escareño, Oscar Arrieta

**Affiliations:** 1 Laboratorio de Medicina Personalizada de la Unidad de Oncología Torácica, Instituto Nacional de Cancerología (INCan), Mexico City 14080, Mexico; rubenrb@comunidad.unam.mx (R.R.-B.); nhernandezp@incan.edu.mx (N.Y.H.-P.); 2 Programa de Doctorado en Ciencias Biomédicas, Facultad de Medicina, Universidad Nacional Autónoma de México (UNAM), Mexico City 04510, Mexico; 3 Departmento de Patología, Instituto Nacional de Cancerología (INCan), Mexico City 14080, Mexico; ccavos@incan.edu.mx; 4 Departamento de Oncología Médica-Tumores Mamarios, Instituto Nacional de Cancerología (INCan), Mexico City 14080, Mexico; pcabrerag@incan.edu.mx (P.C.-G.); cynthia.villarreal@tecsalud.mx (C.V.-G.); 5 Bio Assisted Sequencing Environment National Sequencing Laboratory (Tec-BASE), School of Engineering and Sciences, Tecnologico de Monterrey, Monterrey 64849, Mexico; gerardo.alanisf@tec.mx; 6 Centro de Investigación sobre Enfermedades Infecciosas, Instituto Nacional de Salud Pública, Cuernavaca 62100, Mexico; everardo.gutierrez@insp.edu.mx; 7 Centro de Investigación de Enfermedades Infecciosas, Instituto Nacional de Enfermedades Respiratorias, Mexico City 14080, Mexico; santiago.avila@cieni.org.mx (S.Á.-R.); margarita.matias@cieni.org.mx (M.M.-F.); gustavo.reyesteran@salud.gob.mx (G.R.-T.); 8 Unidad de Investigación Biomédica en Cáncer, INCan-Instituto de Investigaciones Biomédicas, UNAM, Mexico City 14080, Mexico; jdiazchavez03@comunidad.unam.mx; 9 Centro de Cáncer de Mama, Hospital Zambrano Hellion, Tecnologico de Monterrey, Monterrey 66278, Mexico; 10 Laboratorio de Medicina Traslacional, Instituto Nacional de Cancerología (INCan), Mexico City 14080, Mexico; aortegag@incan.edu.mx; 11 Thoracic Oncology Unit, Department of Thoracic Oncology, Instituto Nacional de Cancerología (INCan), Mexico City 14080, Mexico; luis.laram@incmnsz.mx; 12 School of Engineering and Sciences, Tecnologico de Monterrey, Epigmenio González 500, San Pablo, Santiago de Querétaro 76130, Mexico; 13 Computational Genomics and Integrative Biology, National Institute of Genomic Medicine (INMEGEN), Periférico Sur 4809 Arenal Tepepan, Mexico City 14610, Mexico

## Text Correction

A Fine–Gray analysis was missing in the original version [1]. A correction has now been made to *Materials and Methods, Section 2.7: Statistical Analyses*, and to *Results, Section 3.9: Clinical Variables and Outcomes*, where the final paragraph describing the competing risk analysis has been added. Also, Table 4 has been included to effectively represent the results of the Fine–Gray analysis.

The corrected paragraph for *Section 2.7* (*Statistical Analyses*) is given below:

The summary statistics, including means, medians, ranges, and standard deviations, were calculated for continuous variables; the categorical variables were summarized as proportions and confidence intervals (95% CIs). Significant differences among continuous variables for non-parametric distributions were assessed using the Mann–Whitney U test. The Chi-square test or Fisher’s exact test was used to determine statistically significant differences among categorical variables. The PFS and OS were analyzed using the Kaplan–Meier method, while the log-rank test evaluated the differences between subgroups. Univariable and multivariable analyses of the PFS and OS were performed using the Cox proportional hazards model. In addition, because death may preclude observation of progression, we conducted competing risks analyses with progression as the event of interest and death as the competing event. Cumulative incidence functions were estimated for each subgroup and compared using Gray’s test, and subdistribution hazard ratios (SHRs) with 95% CIs were derived from Fine–Gray regression (univariable and multivariable), adhering to the same two-sided significance threshold (*p* < 0.05). Statistical significance was predetermined at a *p*-value < 0.05 based on a two-sided test. The analyses were performed using SPSS version 26 (IBM Company, Armonk, NY, USA) and R statistical software version 4.1.1 (GPL Technologies, Burbank, CA, USA). Competing risks analyses were performed in R (package cmprsk).

The new content added in second paragraph for *Section 3.9* (*Clinical Variables and Outcomes*) is given below:

Using the pooled M and UNS tumors as the reference, the immunomodulatory (IM) subtype showed an sHR of 0.25 (95 % CI 0.08–0.81, *p* = 0.021). All other subtypes mirrored the non-significant behavior reported in the KM and multivariable Cox models (Table 4).

The new Table 4 has been provided above.

The authors apologize for any inconvenience caused and state that the scientific conclusions remain unaffected. This correction was approved by the Academic Editor. The original publication has also been updated.

## Figures and Tables

**Table 4:** Competing risk analysis.

TNBC Molecular Subtype	Sub-Distribution Hazard Ratio (SHR)	Robust s.e.	z-Score	*p*-Value	95% CI (Lower)	95% CI (Upper)
BL1	1.0238	0.5039	0.05	0.962	0.3901	2.6865
BL2	1.3908	0.7935	0.58	0.563	0.4546	4.2549
IM	0.2545	0.1511	−2.31	0.021	0.0795	0.8146
LAR	1.3317	1.0144	0.38	0.707	0.2992	5.9265
MSL	1.047	0.5395	0.09	0.929	0.3813	2.8747
M	1.0000 ^†^	—	—	—	—	—
UNS	1.0000 ^†^	—	—	—	—	—

^†^ Mesenchymal (M) and unclassified (UNS) subtypes served jointly as the reference category and were omitted by the model software; their SHR is therefore fixed at 1.
