# Correction: Rodríguez-Bautista et al. Immune Milieu and Genomic Alterations Set the Triple-Negative Breast Cancer Immunomodulatory Subtype Tumor Behavior. *Cancers* 2021, *13*, 6256

**DOI:** 10.3390/cancers18101627

**Published:** 2026-05-18

**Authors:** Rubén Rodríguez-Bautista, Claudia H. Caro-Sánchez, Paula Cabrera-Galeana, Gerardo J. Alanis-Funes, Everardo Gutierrez-Millán, Santiago Ávila-Ríos, Margarita Matías-Florentino, Gustavo Reyes-Terán, José Díaz-Chávez, Cynthia Villarreal-Garza, Norma Y. Hernández-Pedro, Alette Ortega-Gómez, Luis Lara-Mejía, Claudia Rangel-Escareño, Oscar Arrieta

**Affiliations:** 1 Laboratorio de Medicina Personalizada de la Unidad de Oncología Torácica, Instituto Nacional de Cancerología (INCan), Mexico City 14080, Mexico; rubenrb@comunidad.unam.mx (R.R.-B.); nhernandezp@incan.edu.mx (N.Y.H.-P.); 2 Programa de Doctorado en Ciencias Biomédicas, Facultad de Medicina, Universidad Nacional Autónoma de México (UNAM), Mexico City 04510, Mexico; 3 Departmento de Patología, Instituto Nacional de Cancerología (INCan), Mexico City 14080, Mexico; ccavos@incan.edu.mx; 4 Departamento de Oncología Médica-Tumores Mamarios, Instituto Nacional de Cancerología (INCan), Mexico City 14080, Mexico; pcabrerag@incan.edu.mx (P.C.-G.); cynthia.villarreal@tecsalud.mx (C.V.-G.); 5 Bio Assisted Sequencing Environment National Sequencing Laboratory (Tec-BASE), School of Engineering and Sciences, Tecnologico de Monterrey, Monterrey 64849, Mexico; gerardo.alanisf@tec.mx; 6 Centro de Investigación sobre Enfermedades Infecciosas, Instituto Nacional de Salud Pública, Cuernavaca 62100, Mexico; everardo.gutierrez@insp.edu.mx; 7 Centro de Investigación de Enfermedades Infecciosas, Instituto Nacional de Enfermedades Respiratorias, Mexico City 14080, Mexico; santiago.avila@cieni.org.mx (S.Á.-R.); margarita.matias@cieni.org.mx (M.M.-F.); gustavo.reyesteran@salud.gob.mx (G.R.-T.); 8 Unidad de Investigación Biomédica en Cáncer, INCan-Instituto de Investigaciones Biomédicas, UNAM, Mexico City 14080, Mexico; jdiazchavez03@comunidad.unam.mx; 9 Centro de Cáncer de Mama, Hospital Zambrano Hellion, Tecnologico de Monterrey, Monterrey 66278, Mexico; 10 Laboratorio de Medicina Traslacional, Instituto Nacional de Cancerología (INCan), Mexico City 14080, Mexico; aortegag@incan.edu.mx; 11 Thoracic Oncology Unit, Department of Thoracic Oncology, Instituto Nacional de Cancerología (INCan), Mexico City 14080, Mexico; luis.laram@incmnsz.mx; 12 School of Engineering and Sciences, Tecnologico de Monterrey, Epigmenio González 500, San Pablo, Santiago de Querétaro 76130, Mexico; 13 Computational Genomics and Integrative Biology, National Institute of Genomic Medicine (INMEGEN), Periférico Sur 4809 Arenal Tepepan, Mexico City 14610, Mexico

**Errors in Sections 2.4, 2.7 and 3.2**

In the original publication [1], several minor textual and tabular transcription issues and an ambiguous phrasing were identified in Sections 2.4, 2.7, and 3.2. The corrected text appears below.

**Error in Section 2.4: Classification of TNBC Patients in IM and Non-IM Subtypes**

The sentence “All 68 samples were inserted into the algorithm, which classified 55 samples into one of the aforementioned subtypes; in total, 11 samples were labeled as unclassified.” has been corrected to “All 68 samples were inserted into the algorithm, which classified 55 samples into one of the aforementioned subtypes; in total, 13 samples were labeled as unclassified.”

**Error in Section 2.7: Statistical Analyses**

The software attribution “R statistical software version 4.1.1 (GPL Technologies, Burbank, CA, USA)” has been corrected to “R statistical software version 4.1.1 (R Core Team, Vienna, Austria)”.

**Error in Section 3.2: Treatment Management of TNBC Patients**

The sentence “Out of the cohort of 57 patients with clinical stage III disease, 70.2% did not respond to treatment.” has been corrected to “Out of the cohort of 57 patients with clinical stage III disease, 40 (70.2%) had at least one high-risk pathological feature (positive axillary lymph nodes, histologic grade 3, Ki-67 index > 20% or lymphovascular invasion)”.

The authors state that the scientific conclusions are unaffected. This correction was approved by the Academic Editor. The original publication has also been updated.
