# Supplementary material for: Immune Milieu and Genomic Alterations Set the Triple-Negative Breast Cancer Immunomodulatory Subtype Tumor Behavior
Source: Cancers (Basel). 2021 Dec 13;13(24):6256. doi: 10.3390/cancers13246256 (PMC8699570; doi:10.3390/cancers13246256)
Supplement: Supplementary file 1 [file cancers-13-06256-s001.zip › cancers-1494028-supplementary.pdf]

Supplementary Materials

Immune Milieu and Genomic Alterations Set the Triple-Negative Breast Cancer Immunomodulatory Subtype Tumor Behavior

Rubén Rodríguez-Bautista, Claudia H. Caro-Sánchez, Paula Cabrera-Galeana, Gerardo J. Alanis-Funes, Everardo Gutierrez-Millán, Santiago Ávila-Ríos, Margarita Matías-Florentino, Gustavo Reyes-Terán, José Díaz-Chávez, Cynthia Villarreal-Garza, Norma Y. Hernandez-Pedro, Alette Ortega-Gómez, Luis Lara-Mejía, Claudia Rangel-Escareño and Oscar Arrieta

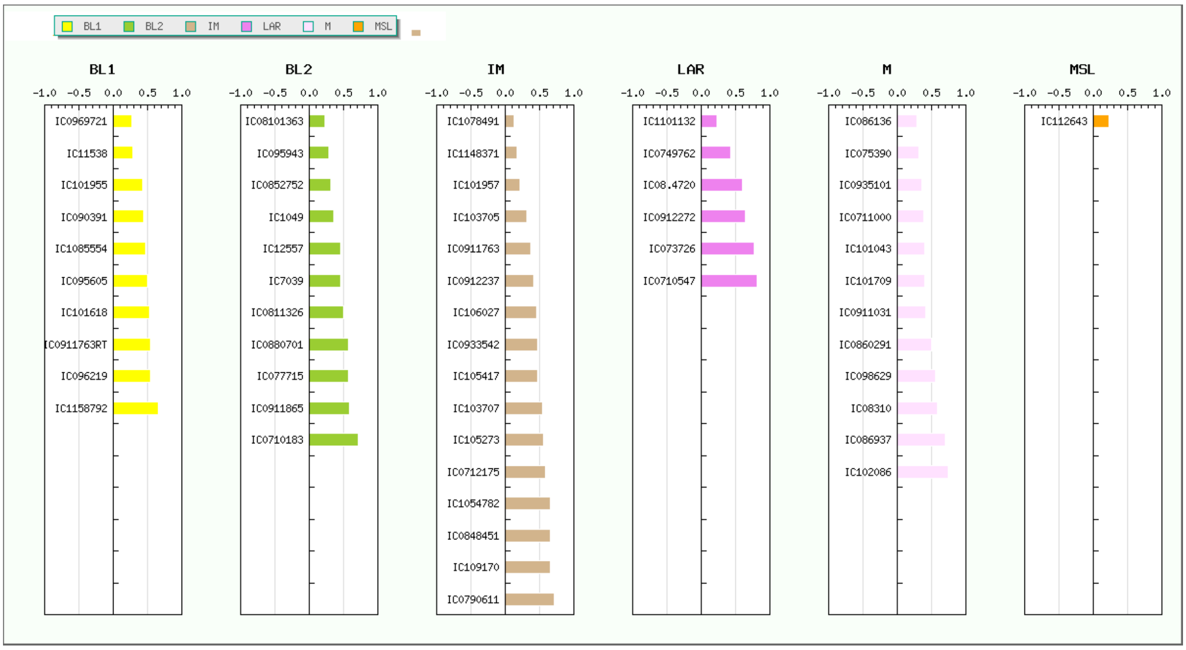

Figure S1. Distributions of the Vanderbilt subtypes in Mexican women with triple-negative breast cancer (TNBC) (n = 68): basal-like 1 (BL1), basal-like 2 (BL2), immunomodulatory (IM), luminal androgen receptor (LAR), mesenchymal (M), and mesenchymal stem-like (MSL). Another 11 samples could not be identified by this software with a significant confidence level.

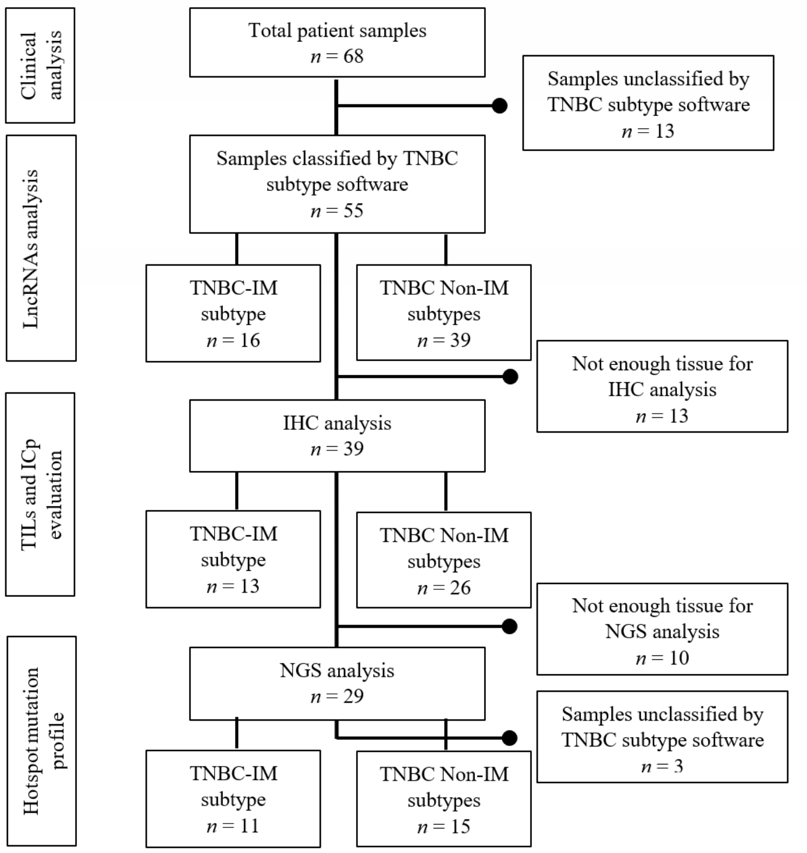

Figure S2. Strobe flow diagram. TNBC: Triple negative breast cancer; IM: immunomodulatory; IHC: immunohistochemistry; NGS: next generation sequencing; long non-coding RNAs (lncRNAS); TILS: Tumor infiltrating lymphocytes; ICp: immune checkpoints.

Table S1. Gene content of cancer panel.

**Genes**
AKT1 (3), *ALK* (21–25), *BRAF* (11, 15), *CDK4* (2), *CDKN2A* (1 *, 2, 3), *CTNNB1* (3), *DDR2* (18), *DICER1* (24, 25), *EGFR* (18–21), *ERBB2* (8, 17, 20), *ERBB4* (10, 12), *FBXW7* (8–12), *FGFR1* (13, 15), *FGFR2* (7, 12, 14), *FGFR3* (7, 9, 14, 16), *FOXL2* (1 *), *GNA11* (4, 5), *GNAQ* (4, 5), *GNAS* (8), *H3F3A* (2 *), *H3F3B* (2 *), *HIST1H3B* (1), *HRAS* (2–4), *IDH1* (4), *IDH2* (4), *KIT* (8–11, 13, 17, 18), *KRAS* (2–4), *MAP2K1* (2, 3), *MET* (2, 14–20), *MYOD1* (1), *NRAS* (2–4), *PDGFRA* (12, 14, 18), *PIK3CA* (2 *, 3, 6 *, 8, 10, 21), *PTPN11* (3), *RAC1* (3), *RAF1* (7, 10, 12, 13 *, 14 *, 15 *), *RET* (11, 13, 15, 16), *ROS1* (38 *, 41 *), *SF3B1* (15–17), *SMAD4* (8–12), *TERT* (promoter *, 1 *, 8 *, 9 *, 13 *), *TP53* (full coding region)

Exon number in parentheses, * Hotspots only.

Table S2. Type of recurrence sites of stage III patients (*n* = 57).

**Recurrence**	**%(*n*/*n*)**
No	29.8 (17/57)
Yes	70.2 (40/57)
CNS recurrence	43.9 (25/57)
Metastases Other Sites no CNS	
Visceral	29.8 (17/57)
No Visceral	5.3 (3/57)
Both	14.0 (8/57)
Metastases to Lung	36.8 (21/57)
Metastases to Liver	19.3 (11/57)
Metastases to Bone	21.1 (12/57)

CNS: Central Nervous System.

Table S3. Differential gene expression analysis coding and long non-coding regions.

Gene ID	LOGFC	AVEEXPR	*t*-Test	*p* Value	ADJ. *p* Value	B
FDCSP	2.943361527	6.850946853	3.955305312	0.000208904	0.01170946	0.515853226
IGKC	2.24664711	7.356507915	3.954025916	0.000209787	0.01173637	0.511982038
IGHM	2.231013275	6.286047314	3.847805474	0.000297036	0.01551561	0.193058658
IGKC	2.123936034	8.079436342	3.918282083	0.000235952	0.012891516	0.404112859
IGHA1	2.103705005	5.952013419	4.292874769	6.72×10^{-5}	0.005139488	1.56053933
IGKC	2.069324519	8.551306661	4.440568321	4.04×10^{-5}	0.003517919	2.031150831
IGHG1	2.013379674	7.688921093	4.268084854	7.31×10^{-5}	0.00546486	1.482316947
IGHV4-31	1.982978135	8.208467165	4.08699288	0.000134883	0.008346984	0.917983833
IGLV1-44	1.981639885	3.70740469	4.957223829	6.45×10^{-6}	0.00092192	3.732406847
IGHG1	1.96910446	9.086445794	4.905015932	7.79×10^{-6}	0.001037077	3.55701903
IGKC	1.938970572	6.809754967	4.620531925	2.15×10^{-5}	0.002295999	2.614586371
CXCL13	1.818226308	5.593121881	6.145128678	7.61×10^{-8}	7.92×10^{-5}	7.871888858
IGLL5	1.785391259	10.22665725	4.951568066	6.59×10^{-6}	0.000931851	3.71337264
IGKV≅	1.745843704	7.592397044	4.980519724	5.93×10^{-6}	0.000860145	3.810894316
CXCL9	1.731316043	7.966204912	4.261334825	7.48×10^{-5}	0.005535427	1.461057195
IGKC	1.695100885	6.158572658	4.898921853	7.97×10^{-6}	0.001050526	3.536593105
IGKC	1.653521857	10.01740769	3.907324131	0.000244584	0.013275411	0.3711542
IGKV3D-15	1.649032155	8.347893953	4.799719601	1.14×10^{-5}	0.001381514	3.205503434
TRAJ17	1.645074727	5.680195043	5.019889742	5.14×10^{-6}	0.000764423	3.943849061
IGHG1	1.578146078	6.779645809	4.255736544	7.63×10^{-5}	0.00558278	1.443437827
IGLV3-25	1.567399741	8.841218986	3.807293988	0.000338748	0.017111742	0.072741306
CXCL10	1.549755728	8.615580185	4.207080894	9.00×10^{-5}	0.006305284	1.290800526
IGK	1.533307511	9.65561248	4.459600572	3.78×10^{-5}	0.003358301	2.092347901
GBP4	1.467080145	7.179058901	4.890195916	8.22×10^{-6}	0.001069571	3.507363001
IGKC	1.462262791	9.112632218	4.133286272	0.000115473	0.007430553	1.061031265
YME1L1	1.454346779	6.020678752	6.511737909	1.86×10^{-8}	4.52×10^{-5}	9.187052419
CD38	1.45006825	6.098312657	5.997847749	1.34×10^{-7}	9.73×10^{-5}	7.346951261
IRF4	1.442298063	6.352035066	5.180562042	2.86×10^{-6}	0.000491682	4.490319118
FCRL5	1.434365642	5.104564812	4.892925942	8.14×10^{-6}	0.00106385	3.516505848
IGHM	1.408334848	7.384230397	5.363446283	1.45×10^{-6}	0.000353343	5.119307099
CD79A	1.398772653	6.883134059	5.902172681	1.92×10^{-7}	0.000123025	7.007260681
CXCR2P1	1.384830625	6.064015095	6.05509216	1.07×10^{-7}	8.67×10^{-5}	7.550705343
PIM2	1.383637253	7.001465215	5.567678968	6.79×10^{-7}	0.000232898	5.829458072
TRAV12-2	1.375753055	5.361220858	5.097362895	3.88×10^{-6}	0.00062071	4.206589283
YME1L1	1.370614713	4.836545567	5.825355882	2.57×10^{-7}	0.000138742	6.735358162
IGHG1	1.351807848	5.847076781	4.467101487	3.68×10^{-5}	0.003291861	2.116500313
UBD	1.335481789	7.503789199	4.853240859	9.39×10^{-6}	0.001184665	3.383797741
TENT5C	1.334892171	7.181763181	4.605384024	2.27×10^{-5}	0.002378397	2.565076177
FAM30A	1.265925483	6.835648924	5.825294045	2.57×10^{-7}	0.000138742	6.735139598
IL2RG	1.255929871	7.464312792	6.776048603	6.69×10^{-9}	2.36×10^{-5}	10.14048779
CCL19	1.22677208	7.651253468	3.861529069	0.00028406	0.014944919	0.233984184
IGLC1	1.219948285	7.607714531	4.548842393	2.77×10^{-5}	0.002734128	2.380911705
SLAMF7	1.213199925	6.574147652	6.280428912	4.53×10^{-8}	5.79×10^{-5}	8.356010201
MZB1	1.197273079	7.35465191	4.944604621	6.75×10^{-6}	0.000941908	3.689948804
TRAV20	1.165609208	3.733066918	5.33860379	1.60×10^{-6}	0.000360407	5.03346172
SLAMF6	1.152327487	4.772127079	5.888984425	2.02×10^{-7}	0.000123025	6.960524665
TRAV20	1.130564502	5.911463894	6.07646435	9.90×10^{-8}	8.56×10^{-5}	7.626869084
GBP5	1.118917883	6.130462332	4.805756945	1.11×10^{-5}	0.001366469	3.225575643
LAX1	1.114841438	5.691522904	5.47767354	9.51×10^{-7}	0.000269933	5.515558511
ANKRD44-IT1	1.114619595	5.398345928	7.186071885	1.36×10^{-9}	2.14×10^{-5}	11.62370392
OR6C4	1.099240147	6.444723972	5.30191346	1.83×10^{-6}	0.000394399	4.906901709
YME1L1	1.096860683	5.04075177	4.765745683	1.28×10^{-5}	0.001540635	3.092743426
YME1L1	1.093800838	4.617918367	3.811595136	0.000334065	0.016933924	0.085480333
GPR174	1.093060811	4.102078365	6.292250112	4.33×10^{-8}	5.79×10^{-5}	8.398384236
SEL1L3	1.090322848	7.044401313	5.533272873	7.73×10^{-7}	0.000250206	5.709297909
CCR2	1.078606635	6.115620598	5.275981805	2.01×10^{-6}	0.000407161	4.817619124
VCAM1	1.065525166	6.15650004	4.501200371	3.27×10^{-5}	0.003083731	2.226533783
BTG2	1.054206604	9.076613637	5.35724828	1.49×10^{-6}	0.000356297	5.097877918
IGKC	1.039223904	7.412084324	4.303013122	6.49×10^{-5}	0.005084913	1.592595057
YME1L1	1.037853606	6.770696289	4.975084582	6.05×10^{-6}	0.000872718	3.792570132
TRDV2	1.037646389	3.670155434	5.215321289	2.51×10^{-6}	0.000458124	4.609318714
MIR142	1.033700489	4.774926725	4.763015112	1.30×10^{-5}	0.001548397	3.083694906

CCL5	1.033222203	7.59358146	5.876897681	2.12×10^{-7}	0.000123025	6.917711683
IFNG	1.029312304	4.65923508	6.399562831	2.87×10^{-8}	5.61×10^{-5}	8.783557791
YME1L1	1.028751756	3.414385854	5.601535183	5.98×10^{-7}	0.000215255	5.94789292
CD2	1.027183095	7.216852064	5.808298453	2.74×10^{-7}	0.000138742	6.675088041
SLFN12L	1.017642293	5.541069054	6.150462534	7.46×10^{-8}	7.92×10^{-5}	7.890941865
IL23A	1.013598461	5.190110719	4.729454739	1.46×10^{-5}	0.001689798	2.972659022
TRAV12-2	1.012090486	5.930724244	4.299928847	6.56×10^{-5}	0.005084913	1.582839135
ITGAL	1.008762873	6.514419207	6.115592128	8.52×10^{-8}	8.28×10^{-5}	7.766432672
IGKC	1.008642719	5.57773397	5.540534135	7.52×10^{-7}	0.000249032	5.734640302
BCL2A1	1.007547625	5.561778583	4.024974657	0.000165882	0.009729212	0.727703982
YME1L1	1.006633651	4.265723623	4.872486364	8.76×10^{-6}	0.001116699	3.448102231
CTSS	1.005838028	7.306051985	5.04659995	4.66×10^{-6}	0.000708075	4.03426944

LOFFC: Log Fold chance; AVEEXP: average expression; ADJ: adjusted.

Table S4. Clinical and pathological characteristics of TNBC patients with IM and non-IM subtypes.

Clinical Characteristics	IM	Non-IM	*p*-Value
	%(*n*/*n*)	%(*n*/*n*)	
Hormonal Status			
Premenopausal	73.3(11/15)	45.2(19/42)	-
Postmenopausal	26.7(4/15)	54.8(23/42)	0.057
Vascular Invasion			
Yes	66.7(10/1d5)	33.3 (14/42)	-
No	33.3(5/15)	40.4(28/42)	0.026 *
PCR			
Yes	45.5(5/11)	23.3(7/30)	-
No	54.5(6/11)	76.6(23/30)	0.160
Recurrence			
Yes	33.3 (5/15)	76.2(32/42)	-
No	66.7 (10/15)	23.8 (10/42)	0.004 *
Lung Metastases			
Yes	0(0/15)	50 (21/42)	-
No	100 (15/15)	50 (21/42)	<0.001 *
Liver Metastases			
Yes	0 (0/15)	26.2 (11/42)	-
No	100 (15/15)	73.8 (31/42)	0.023 *
Bone Metastases			
Yes	6.7 (1/15)	26.2 (11/42)	-
No	93.3 (14/15)	73.8 (31/42)	0.106
CNS Metastases			
Yes	20 (3/15)	47.6 (20/42)	-
No	80 (12/15)	52.4 (22/52)	0.022 *

Bivariate analysis by chi-square/Fisher exact for the association between the dependent variable (molecular subtypes) and independent variables (clinical and pathological variables). PCR: complete pathological response; CNS: central nervous system; EGFR: epidermal growth factor receptor; * $p < 0.05$.

Table S5. Univariate and multivariate analysis with RFS and OS of TNBC patients.

Variable	RFS				OS			
	Univariate Analysis		Multivariate Analysis		Univariate Analysis		Multivariate Analysis	
	**HR (95%CI)**	***p*-value**	**HR (95% CI)**	***p*-value**	**HR (95%CI)**	***p*-value**	**HR (95%CI)**	***p*-value**
Stage								
III	-		-		Reference	-	-	
IV					2.4 (1.2–4.7)	0.014 *		
Mastectomy								
Done	Reference	-	Reference	-	Reference	-	Reference	-
Not Done	2.7 (1.3–5.5)	0.007 *	3.6 (1.6–7.7)	0.001 *	2.3(1.3–4.1)	0.004 *	1.9(1.1–3.5)	0.037 *
Recurrence								
No	Reference		-		Reference	-	-	
Yes	36.8 (4.9–276.4)	<0.001 *			36.8(4.9–276.4)	<0.001 *		
RT to SNC								
No	Reference	-	-	-	Reference	-	Reference	-
Yes	3.73 (1.9–7.3)	<0.001 *	2.6 (1.3–5.5)	0.007 *	3.5(1.9–6.3)	<0.001 *	2.4(1.3–4.5)	0.008 *
Metastasis								
None	Reference	-	-		Reference	-	-	
Visceral	3.2 (1.5–6.8)	0.004 *			2.6 (1.4–5.1)	0.004 *		
Non Visceral	3.7 (1.03–13.5)	0.045 *			1.9 (0.5–6.5)	0.328		
Both	3.3 (1.2–9)	0.019 *			2.9(1.2–7.1)	0.017 *		
Immunomodulator								
Non-IM	Reference	-	Reference	-	Reference	-	Reference	-
IM	0.3 (0.1–0.8)	0.011 *	0.3 (0.1–0.9)	0.031 *	0.3(0.1–0.7)	0.007 *	0.4(0.2–0.9)	0.038 *
Bone Metastasis								
No	Reference	-	-		Reference	-	-	
Yes	2.01 (0.96–4.24)	0.065			1.6 (0.9–3)	0.124		
Lung Metastases								
No	Reference	-	-		Reference	-	-	
Yes	1.91 (1–3.7)	0.054			2.2 (1.2–3.8)	0.007 *		
Liver Metastases								
No	Reference	-	-		Reference	-	-	
Yes	1.8 (0.84–3.83)	0.13			2.3(1.3–4.1)	0.006 *		
CNS Metastases								
No	Reference	-	-		Reference	-	-	
Yes	4.74 (2.3–9.8)	<0.001 *			4.5 (2.3–8.5)	<0.001 *		

TNBC: Triple negative breast cancer; rfs: recurrence-free survival; OS: overall survival (OS); HR: hazard ratio; RT: radiotherapy; CNS: central nervous system; IM: immunomoduadory (IM); * $p < 0.05$.

© 2021 by the authors. Licensee MDPI, Basel, Switzerland. This article is an open access article distributed under the terms and conditions of the Creative Commons Attribution (CC BY) license (http://creativecommons.org/licenses/by/4.0/).
